# Investigating off-Hugoniot states using multi-layer ring-up targets

**DOI:** 10.1038/s41598-020-68544-8

**Published:** 2020-08-06

**Authors:** D. McGonegle, P. G. Heighway, M. Sliwa, C. A. Bolme, A. J. Comley, L. E. Dresselhaus-Marais, A. Higginbotham, A. J. Poole, E. E. McBride, B. Nagler, I. Nam, M. H. Seaberg, B. A. Remington, R. E. Rudd, C. E. Wehrenberg, J. S. Wark

**Affiliations:** 1 Department of Physics, Clarendon Laboratory, University of Oxford, Parks Road, Oxford, OX1 3PU UK; 2 Los Alamos National Laboratory, Bikini Atoll Road, SM-30, Los Alamos, NM 87545 USA; 3 Atomic Weapons Establishment, Aldermaston, Reading, RG7 4PR UK; 4 Department of Chemistry, Massachusetts Institute of Technology, Cambridge, MA 02139 USA; 5 Department of Physics, University of York, Heslington, York, YO10 5DD UK; 6 SLAC National Accelerator Laboratory, Menlo Park, CA 94025 USA; 7 Lawrence Livermore National Laboratory, Livermore, CA 94550 USA

**Keywords:** Condensed-matter physics, Techniques and instrumentation

## Abstract

Laser compression has long been used as a method to study solids at high pressure. This is commonly achieved by sandwiching a sample between two diamond anvils and using a ramped laser pulse to slowly compress the sample, while keeping it cool enough to stay below the melt curve. We demonstrate a different approach, using a multilayer ‘ring-up’ target whereby laser-ablation pressure compresses Pb up to 150 GPa while keeping it solid, over two times as high in pressure than where it would shock melt on the Hugoniot. We find that the efficiency of this approach compares favourably with the commonly used diamond sandwich technique and could be important for new facilities located at XFELs and synchrotrons which often have higher repetition rate, lower energy lasers which limits the achievable pressures that can be reached.

## Introduction

For the past century, there has been considerable interest in studying material properties at high pressure^1–6^. Traditional static techniques for generating these states, such as using diamond anvil cells (DACs), are limited by the strength of the diamonds^7^. While the use of double stage DACs has achieved pressures in excess of 1 TPa^8^, to reach higher pressures, laser compression is required. While much work has been done using lasers to shock materials, this process is highly entropic and will result in the sample being heated until eventually, the sample will melt. For most metals, this is usually below 300 GPa^9–14^. To push the pressure beyond this point, while keeping the sample solid, ramp compression is required to keep the material closer to an isentrope. These techniques are often paired with in situ X-ray diffraction which provides measurements of density and structure, and which has previously been proven in laser-shock experiments^6,10,11,15–29^.

The standard method to perform these high pressure diffraction measurements on quasi-isentropically compressed material was developed by Rygg and coworkers^30^, where they sandwiched a thin sample between two diamond anvils and then used a ramped laser pulse to slowly compress the sample over several nanoseconds. A quasi-monochromatic X-ray backlighter from a laser-plasma source could then be used to record X-ray diffraction measurements at peak compression. This method has been used at the Omega laser facility^31^ to ramp compress Al to 475 GPa^32^ and Mo, Sn and Fe–Si alloys to above 1 TPa^33–35^. While there has been significant success using this approach, the advent of X-ray Free Electron Lasers (XFELs) and in particular high energy density beamlines that pair these intense X-ray sources with nanosecond lasers^36–38^, has sparked significant interest in performing ramp compression experiments using these facilities^39^. To date, these FELs use much smaller optical lasers, but can still reach significant pressures, as the narrow X-ray beam (typically 10–50 μm) means that a much smaller volume of sample needs to be compressed. XFELs offer a number of benefits that make them attractive for these type of experiments. The smaller laser systems allow for much higher repetition rates, therefore greatly improving the amount of data that can be gathered. The high intensity and low bandwidth of the XFEL beam allow for the identification of highly complex structures, such as commensurate host-guest phases^10,40^. Lastly, XFELs such the European XFEL and LCLS II will be able to reach very high photon energies (> 20 keV)^39,41^, which allows for much greater filtering to be used in front of detectors, reducing background caused by the ablation plasma from the drive laser and therefore increasing signal-to-noise, as well as allowing a greater volume of reciprocal space to be explored.

These XFEL facilities present different challenges to their large laser facility counterparts. It is therefore reasonable to assume that the diamond sandwich method, which was optimised for these larger facilities, may not be the only viable method for performing off-Hugoniot in situ X-ray diffraction at high pressure. In fact, at these smaller facilities, other techniques may present some advantages. Targets that could be easily mass produced cheaply would be better suited to high repetition rate facilities, where hundreds of targets may be required for a single shift.

We propose a ‘ring-up’ target, where our sample is sandwiched between two higher impedance anvils. This approach, which uses different shock impedance layers to break up a single large shock into several smaller shocks allowing for a cooler compression path, has been previously used in gas gun experiments^42,43^. Simulation work by Aliverdiev and co-workers proposed that this approach could be miniaturised for laser experiments and suggested that by sandwiching a thin Al sample between two thicker Au anvils, off-Hugoniot Al could be created up to pressure of 1 TPa for a laser intensity of $10^{14}\, \mathrm {W\,cm}^{-2}$^44^. While the high impedance mismatch between the Al and Au results in a very efficient way to create high pressures in the Al, the large difference in atomic number between the two materials poses difficulties for an X-ray diffraction experiment, as any diffraction from the sample will be swamped by that of the anvils. Instead, we strike a balance between efficiency and signal-to-noise by choosing a Mo anvil and a Pb sample. This design also has some additional benefits for a proof of principle experiment. Firstly, Mo remains body-centred cubic (BCC) from ambient pressure to over 1 TPa^33^, which simplifies the analysis of the diffraction pattern. Secondly, Pb shock melts at the comparatively low pressure of ~ 50–60 GPa^45,46^ and therefore we can readily test if we are generating off-Hugoniot states by observing diffraction from solid Pb well above this relatively low pressure.

The structure of the paper is as follows. In the next section we discuss simulating a shock propagating through the target using hydrocode simulations. We propose a model using a Mie-Grüneisen equation of state (EOS) to find the pressure and temperature of the Pb sample as it undergoes multiple reverberations. In the results section we discuss an experiment using a Pb ‘ring-up’ target carried out at the Matter in Extreme Conditions instrument (MEC) of the Linac Coherent Light Source (LCLS). In the discussion section we compare the efficiency of this design to diamond sandwich ramp targets and provide our conclusions. Finally in the methods section we discuss how the experiment was performed and how the targets were manufactured.

## Simulations

**Figure 1:**
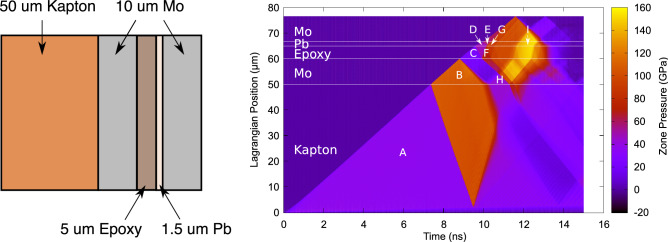
A simulation of the ‘ring-up’ target performed by hydrodynamics code HYADES. The multiple layers of different impedances result in the shock in the ablator being broken up into several smaller shocks in the sample, causing it to follow a cooler compression path. Additionally, the sample undergoes a further increase in pressure due to a rarefaction wave, caused by the shock entering the epoxy layer from the front Mo anvil, reflecting off the compressed Kapton ablator and recompressing the sample. Labels A-I refer to different pressure states in various layers within the target.

To understand the interactions of the different reflections within the sample, the target was modelled using the 1D hydrocode HYADES^47^. The results of one such simulation are shown in Fig. 1. The effect of the multiple different impedance layers results in the incoming shock, formed as the pressure wave steepens up in the Kapton, being broken up into several smaller shocks by the time it reaches the Pb layer. Since the final temperature of the sample is dominated by the magnitude of the initial shock, this results in the sample taking a much cooler compression path. Additionally, at late time, we see a further compression of the sample. This occurs because after the shock wave travels from the first Mo layer into the epoxy, it creates a rarefaction wave that travels back through the Mo anvil and reflects off the Kapton ablator (H), causing an additional compression wave to travel through the target, significantly increasing the peak pressure (I).

**Figure 2:**
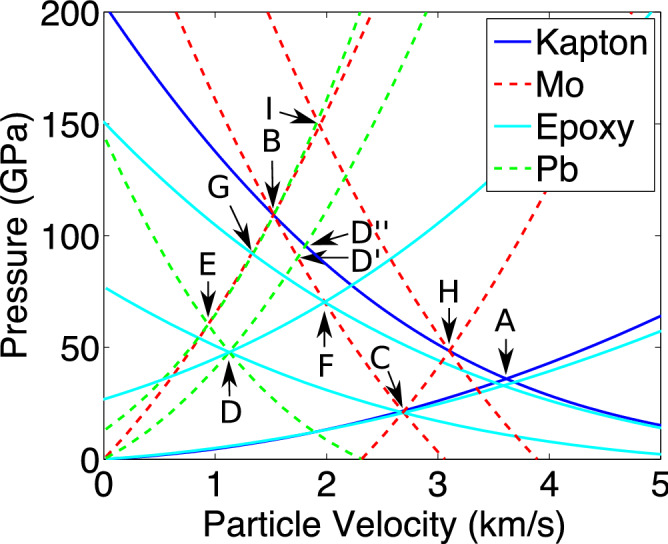
The initial and final pressures in the Pb can be calculated using an impedance matching diagram. The addition of an extra Pb Hugoniot and release isentrope can account for the extra compression due to the reflected release wave recompressing the sample. Labels A-I refer to the P-$\mathrm {u_p}$ states calculated for the corresponding sections in Fig. 1.

Both the initial ‘ring-up’ and the additional compression can be explained by impedance matching using a Pressure-Particle Velocity (P-$\mathrm {u_p}$) diagram (shown in Fig. 2). After the initial shock wave travels through the Kapton (A), each layer will release into the next layer in the target. Depending on the impedance mismatch between the two layers (ie. whether the sample ‘rings up’ or ‘rings down’) the proceeding layer will either follow a secondary Hugoniot or a release isentrope (see Supplementary Materials). To find the pressure of the shock in the new layer, we find where this path crosses the Hugoniot of the next layer. By doing this for each layer (ie. travelling along the path A–B–C–D in Fig. 2), we can find the pressure of the initial shock in the Pb sample, as well as the pressure from the reflected shock from the rear Mo anvil (E). Note that if the target did not contain the epoxy layer, the initial shock would be significantly larger (D’). To find the pressure of the next ring of the Pb, we have to consider the interaction between this reflected shock and the reverberation between the epoxy layer and the front Mo anvil (F), found at position G. We can apply the same analysis to the rarefaction wave travelling back through the front Mo anvil as it reflects off the Kapton ablator (H). Finally, the reflected release wave in the front anvil and the reflected shock from the rear anvil interact to cause an additional compression (I). This final pressure is significantly larger than that attained in a Kapton-Pb shock target using the same laser drive (D”), suggesting that this multilayer technique may be useful for obtaining higher pressures at facilities where laser size or drive noise issues limit the achievable pressures.

**Figure 3:**
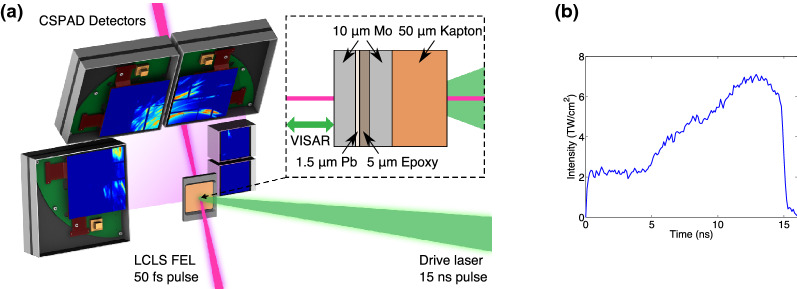
(**a**) A diagram showing the experimental setup. A shock wave is driven by laser ablation into the target package (inset). The shocked sample is interrogated by an X-ray beam, with the resulting diffraction captured on CSPAD detectors. VISAR is used to record the rear surface velocity and breakout time. (**b**) A typical laser pulse.

**Figure 4:**
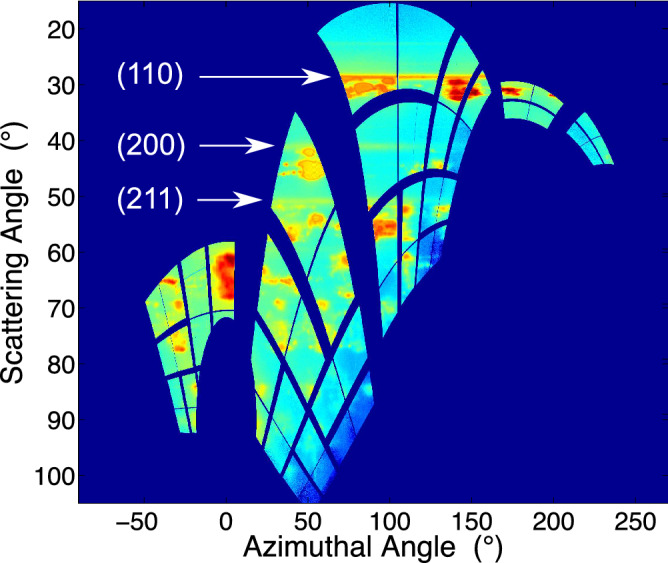
Diffraction data (shown on a log scale) taken at peak compression, warped into spherical coordinates. The highly textured diffraction is from the Mo anvils, whereas the azimuthally more uniform diffraction is from BCC Pb at 150 GPa (labelled in white).

## Results

**Figure 5:**
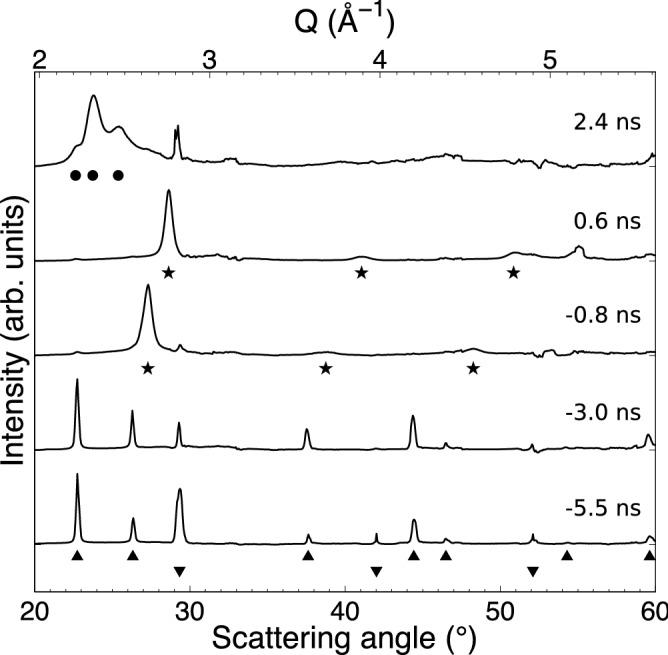
Integrated diffraction profiles recorded at a number of different delays with respect to the breakout of the shock in the rear anvil. Highly textured areas are removed from the integration to reduce the impact of the anvils on the integration, thereby making the diffraction profiles from the relatively untextured sample clearer. The peaks labelled with upward and downward facing triangles correspond to ambient face-centred cubic (FCC) Pb and BCC Mo respectively. Just before and after shock breakout of the rear anvil, BCC Pb lines appear (stars), corresponding to $V/V_0$ of $0.636\pm 0.006$ and $0.548\pm 0.004$ respectively. At late times, a triplet of lines appear (circles) that corresponds to hexagonal close-packed (HCP) Pb with $V/V0 = 0.865\pm 0.02$ and $c/a = 1.65\pm 0.02$.

The experiment was performed at the MEC instrument of the LCLS. A schematic of the experimental setup is shown in Fig. 3. A Nd:glass laser was used to drive a shock through the multilayer target, with the XFEL pulse timed to probe the sample at a different time delay during each shot. Figure 4 shows an example of the diffraction recorded at peak compression, warped into spherical coordinates. Since the target contains both Pb and Mo layers, the data show two diffraction patterns from both materials. However, while the Pb layer is relatively untextured, the Mo was chosen to have a strong texture that does not change significantly under compression, allowing for the two diffraction patterns to be distinguished. By removing highly textured areas (see Supplementary Materials), we produce integrated diffraction profiles where the effect from the anvil diffraction is greatly reduced, thereby making the diffraction profiles from the relatively untextured sample significantly clearer. Figure 5 shows a summary of the data for the same laser intensity at different time delays. While the analysis above had assumed a steady laser drive, the drive profile used in the experiment exhibited a ramp towards the end of the pulse that complicated the analysis. HYADES simulations using laser pulses captured from the experiment were compared with rear surface velocity measurements from VISAR to derive the initial shock and peak pressures reached in the Pb sample layer, which were $19.5\pm 6.5$ GPa and $150\pm 10$ GPa respectively (see Supplementary Materials).

**Figure 6:**
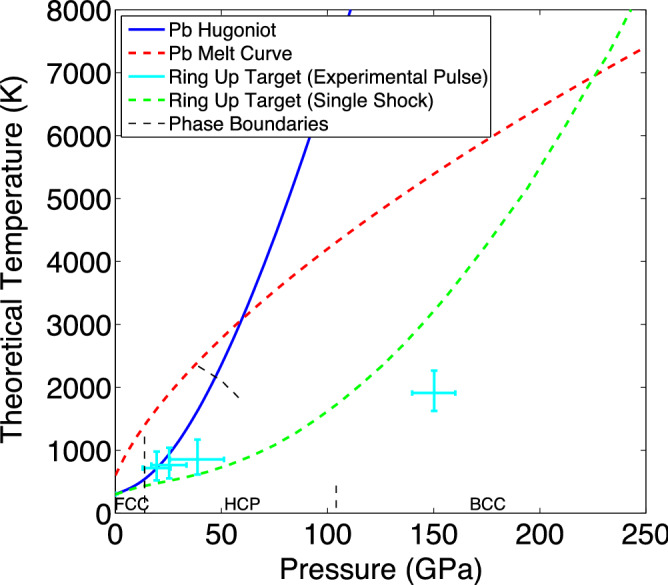
A theoretical P-T plot using a Mie–Grüneisen equation of state for the first, second, third shocks and final state of the Pb sample is shown in cyan. The theoretical Hugoniot temperature for the same EOS is shown in blue, while the melt curve and equilibrium phase boundaries given by Dewaele^45^ are shown in dashed red and dashed black respectively. The loci of final P-T states in the Pb sample using this ‘ring-up’ target design achievable with a single shock is shown in dashed green.

To estimate how far below the Hugoniot temperature the sample might be, we present a theoretical Pressure-Temperature (P-T) diagram using a Mie-Grüneisen equation of state. By using the impedance matching approach described in the previous section, we can find the pressure of the second and third shock in the Pb given an initial shock of 19.5 GPa and then approximate the complex interactions of the subsequent rings and the ramping up of the laser pulse as an isentropic compression up to 150 GPa (see Supplementary Materials). This results in a predicted peak volumetric compression (V/V0) of $0.543\pm 0.01$, which agrees well with the experimentally observed value obtained via diffraction of $0.548\pm 0.004$, giving us confidence in this technique. The corresponding temperature increases are found by modifying an equation given by Meyers^48^ to be valid for reshocked states (see Supplementary Materials). While this approach ignores other energy sources and sinks (such as phase transitions), they are expected to be small compared with other sources of error and therefore can be neglected. Figure 6 shows the different P-T conditions after each successive shock, as well as the final state at maximum compression (cyan line). This is not only much lower in temperature than the theoretical Hugoniot (given by a Mie-Grüneisen equation of state), shown in blue, but is also significantly lower than the green-dashed line representing the achievable P-T states created by using a square laser pulse to shock compress a ‘ring-up’ target, suggesting that it may be possible to create solid matter at pressures even higher than the ~ 200 GPa limit predicted for flat laser pulses. Note that while the ramped laser pulse does provide some off-Hugoniot compression, the majority of the temperature decrease is from the reverberations in the ‘ring-up’ target and that the ramped pulse itself would not provide a cool enough compression path for the Pb to reach 150 GPa without melting (see Supplementary Materials).

**Figure 7:**
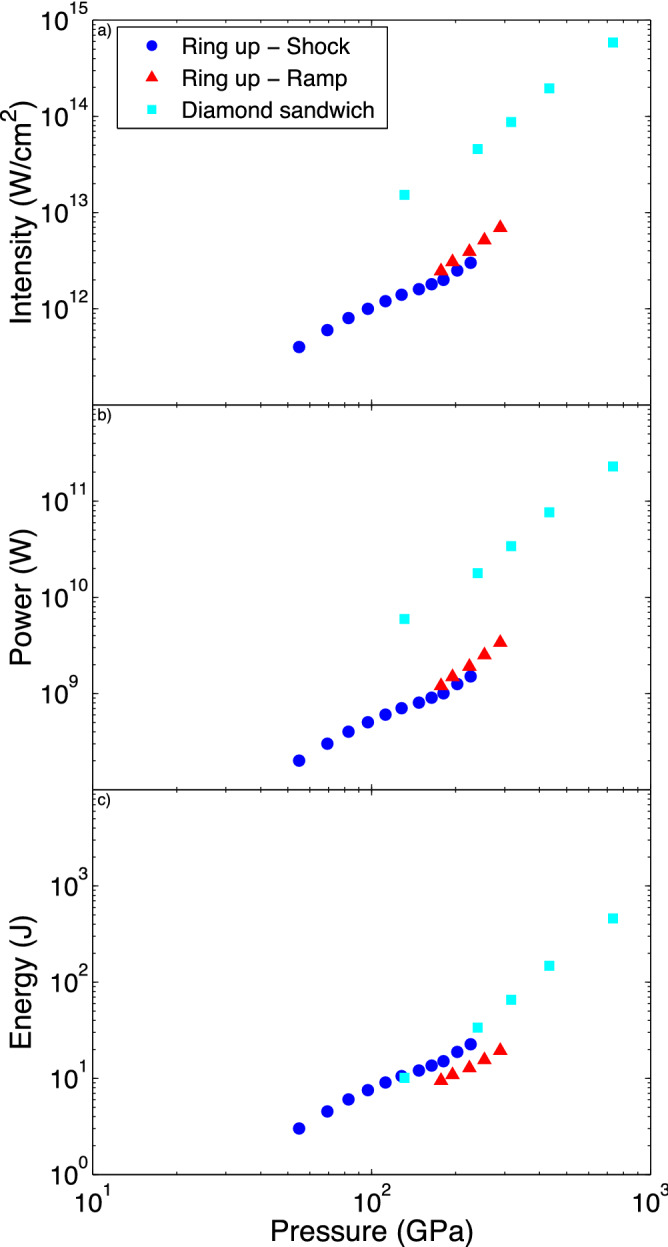
A comparison of how the required: (**a**) intensity, (**b**) power and (**c**) energy varies with peak pressure, for different target/pulse shape types. Only points below the Pb melt curve are shown.

## Discussion

To test the benefits of this design, we compared its performance to that of a diamond sandwich ramp target, via HYADES simulations. The dimensions of the target were chosen based on previous designs used in experiments investigating ramp compressed Sn and Mo performed by Lazicki and Wang respectively^33,34^. The design consists of a 20 μm diamond ablator/pusher a 1.5 μm Pb sample and a 40 μm diamond window. The laser pulse used was a concave ramp (designed to compress the Pb over $~1\,\hbox {ns}$), followed by a $~1 \,\hbox {ns}$ hold. Examples of pulses used are shown in Supplementary Fig. S4.

While the ‘ring-up’ targets were designed to reach off-Hugoniot states without the need of a ramped laser pulse, as alluded to in the previous section, there may be benefits in combining these two techniques. It is often difficult to ramp compress plastic ablators, due to their high compressibility requiring very long pulses to prevent shocking up of the ramp within the ablator. One way to overcome this is by initially shocking the Kapton and then ramp compressing. This is often undesirable as it requires a relatively large initial shock to increase the stiffness of the Kapton, leading to a high initial shock in the sample (due to impedance mismatching), resulting in a higher final temperature. However, as we have demonstrated above, even with a relatively large shock in the ablator, the Pb sample is kept cool enough to remain solid. This allows the target to subsequently be ramp compressed, greatly increasing the pressure in the sample while only resulting in a relatively small increase in temperature.

Depending on the type of experimental facility, peak intensity, peak power and total energy of the drive laser can be the limiting factors that prevent one reaching higher pressures. Figure 7 shows a comparison of the efficiency of the different designs for each of the three parameters. For the case of peak intensity, the pressure reached is independent of the total size of the target, whereas for both power and energy comparisons the total size of the target is important as it will affect both the required spot size and pulse length of the drive laser. To ensure that despite transverse release there is a large enough region of the sample at uniform pressure to use rear surface velocimetry for pressure determination^49^, we have chosen the spots size to be twice the total thickness of the target package + 100 μm (ie. 253 μm and 223 μm for the ‘ring-up’ and diamond sandwich targets respectively). We find that the ‘ring-up’ targets are significantly more efficient in terms of intensity and power (by almost an order of magnitude), whilst also being comparable with the diamond sandwich targets in terms of total energy. Note that this comparison slightly overestimates the performance of the diamond ramp targets, since most laser systems include a frequency doubling or tripling crystal, whose conversion efficiency is greatly decreased during the low intensity initial part of the ramp. While ‘ring-up’ targets will produce higher temperature states than diamond sandwich targets for a given pressure, only points below the Pb melt curve are included in Fig. 7. Accurately measuring this temperature difference due to different compression paths remains experimentally challenging and highlights the importance of the development of in situ temperature diagnostics.

Given these advantages, we believe these targets are well suited to new high repetition rate facilities such as the European XFEL and LCLS II. This design is significantly cheaper than diamond sandwich targets and could be scaled up to meet the large target requirements of these new repetition rate facilities. We envision that these targets could be used to complement diffraction studies using diamond sandwich ramp targets, with the ‘ring-up’ targets used to perform large phase diagram scans, while a smaller number of diamond targets could be used for structure and phase boundary identification. Note that while the data we have presented is at relatively modest pressure, for materials with a higher melting point, much larger compressions are achievable. For example, using this technique with a Fe sample would allow for pressures of above 500 GPa to be reached.

In conclusion, we have demonstrated off-Hugoniot compression of Pb through laser shocking a multi-layer ‘ring-up’ target. Using this design, we reached a pressure of approximately 150 GPa while keeping the Pb solid. By exploiting the difference in texture between the sample and anvils, we are able to distinguish between the two diffraction patterns, allowing for the removal of signal from the anvils. Lastly, we have demonstrated that this approach offers the opportunity to reach higher pressures for a given laser intensity and integrated energy.

## Methods

The experiments were performed at the MEC instrument of the Linac Coherent Light Source (LCLS). A 25 J, 2$\omega$ 15 ns ramped laser pulse was used to dynamically compress the ablator of the target. The drive beam spot was a 250 μm-diameter super-Gaussian, achieved through the use of hybrid phase plates. After a set delay, a 35 μm-diameter 11 keV (0.2% bandwidth) X-ray beam with a 50 fs pulse length was used to interrogate the sample, with the resulting diffraction pattern captured on CSPAD detectors^50^. VISAR was used to determine the breakout time of the free surface and therefore help with timing the X-ray delay to probe the sample at maximum compression. Powder samples of $\mathrm {CeO}_2$ and $\mathrm {LaB}_6$ X-ray standards were used to calibrate the positions and tilts of the detectors and a fluorescent YAG (Yttrium Aluminium Garnet) sample was loaded in order to align the drive lasers with the VISAR laser.

The samples were prepared by first depositing a 1.5 ± 0.1 μm Pb layer on a 10 ± 1.5 μm Mo rolled foil. This was then glued to another 10 ± 1.5 μm Mo rolled foil, using a plastic spacer to ensure a consistent 5 ± 1 μm epoxy layer. A 50 ± 5 μm Kapton ablator was then attached to the second Mo foil.

## Supplementary information

Supplementary Information.
