# Supplementary material for: Investigating off-Hugoniot states using multi-layer ring-up targets
Source: Sci Rep. 2020 Aug 6;10:13172. doi: 10.1038/s41598-020-68544-8 (PMC7413406; doi:10.1038/s41598-020-68544-8)
Supplement: Supplementary file 1 — Supplementary Information. [file 41598_2020_68544_MOESM1_ESM.pdf]

Investigating off-Hugoniot states using multi-layer ring-up targets

D. McGonegle,* P. G. Heighway, and M. Sliwa
Department of Physics, Clarendon Laboratory, University of Oxford, Parks Road, Oxford OX1 3PU, UK

C. A. Bolme
Los Alamos National Laboratory, Bikini Atoll Road, SM-30, Los Alamos, New Mexico 87545, USA

A. J. Comley
Atomic Weapons Establishment, Aldermaston, Reading RG7 4PR, UK

L. E. Dresselhaus-Marais
Department of Chemistry, Massachusetts Institute of Technology, Cambridge, Massachusetts, 02139, USA

A. Higginbotham and A. J. Poole
University of York, Department of Physics, Heslington, York YO10 5DD, UK

E. E. McBride, B. Nagler, I. Nam, and M. H. Seaberg
SLAC National Accelerator Laboratory, Menlo Park, California 94025, USA

B. A. Remington, R. E. Rudd, and C. E. Wehrenberg
Lawrence Livermore National Laboratory, Livermore, CA 94550,USA

J. S. Wark
Department of Physics, Clarendon Laboratory, University of Oxford, Parks Road, Oxford OX1 3PU, UK

CALCULATION OF RESHOCK HUGONIOTS AND RELEASE ISENTROPES

From conservation of mass, momentum and energy we find the Rankine-Hugoniot equations-

$$V_{i+1}/V_i = 1 - \left(u_{p_{i+1}} - u_{p_i}\right) / \left(u_{s\,i+1} - u_{p_i}\right) \quad (1)$$

$$P_{i+1} - P_i = \frac{1}{V_i}\left(u_{s\,i+1} - u_{p_i}\right)\left(u_{p_{i+1}} - u_{p_i}\right) \quad (2)$$

$$E_{i+1} - E_i = \frac{1}{2}\left(P_{i+1} + P_i\right)\left(V_i - V_{i+1}\right) \quad (3)$$

where V, u_p, u_s, P and E are the specific volume, particle velocity, shock velocity, pressure and internal energy respectively. Assuming a linear relationship between particle and shock velocity, $u_s = Cu_p + S$, we can rearrange these equations to find the pressure of the principal Hugoniot is given by-

$$P_H = \frac{C^2\left(V_0 - V_1\right)}{\left[V_0 - S\left(V_0 - V_1\right)\right]^2}. \quad (4)$$

Given a Mie-Grüneisen equation of state, we can relate the pressure and internal energy of a material using the Grüneisen parameter, γ-

$$P_{i+1} - P_H\left(V_{i+1}\right) = \frac{\gamma\left(V_{i+1}\right)}{V_{i+1}}\left(E_{i+1} - E_H\left(V_{i+1}\right)\right) \quad (5)$$

$$E_{i+1} - E_H\left(V_{i+1}\right) = \frac{V_{i+1}}{\gamma\left(V_{i+1}\right)}\left(P_{i+1} - P_H\left(V_{i+1}\right)\right) \quad (6)$$

where the subscript H denotes that these values are calculated on the principal Hugoniot. By subtracting Eq. 6 from Eq. 3, we get-

$$E_H\left(V_{i+1}\right) - E_i = \frac{1}{2}\left(P_{i+1} + P_i\right)\left(V_i - V_{i+1}\right) - \frac{V_{i+1}}{\gamma\left(V_{i+1}\right)}\left(P_{i+1} - P_H\left(V_{i+1}\right)\right) \quad (7)$$

$$E_H\left(V_{i+1}\right) - E_i = \left[\frac{1}{2}\left(V_i - V_{i+1}\right) - \frac{V_{i+1}}{\gamma\left(V_{i+1}\right)}\right]P_{i+1} + \frac{1}{2}P_i\left(V_i - V_{i+1}\right) + \frac{V_{i+1}}{\gamma\left(V_{i+1}\right)}P_H\left(V_{i+1}\right) \quad (8)$$

$$P_{i+1} = \left[E_H\left(V_{i+1}\right) - E_i - \frac{1}{2}P_i\left(V_i - V_{i+1}\right) - \frac{V_{i+1}}{\gamma\left(V_{i+1}\right)}P_H\left(V_{i+1}\right)\right] / \left[\frac{1}{2}\left(V_i - V_{i+1}\right) - \frac{V_{i+1}}{\gamma\left(V_{i+1}\right)}\right], \quad (9)$$

which gives the new pressure (P_{i+1}) as a function of compression (V_{i+1}). To find the new particle velocity ($u_{p_{i+1}}$), we rearrange Eq. 1-

$$1 - V_{i+1}/V_i = \left(u_{p_{i+1}} - u_{p_i}\right) / \left(u_{s\,i+1} - u_{p_i}\right), \quad (10)$$

Material	ρ_0	C	S	γ_0	q	EOS #
Kapton	1.42	1.773	1.45	1.53	1.0	7770
Mo	10.2	5.12	1.25	1.59	1.0	2980
Epoxy	1.185	2.838	1.366	1.53	1.0	7601
Pb	11.346	2.26	1.32	2.74	3.0	3200

TABLE I. The parameters used to model each layer in the target, as well as the SESAME EOS table used for the HYADES simulations.

and then by multiplying this by Eq. 2 we find-

$$(1 - V_{i+1}/V_i)\left(P_{i+1} - P_i\right) = \frac{1}{V_i}\left(u_{p_{i+1}} - u_{p_i}\right)^2 \qquad (11)$$

$$u_{p_{i+1}} = u_{p_i} \pm \left[\left(V_i - V_{i+1}\right)\left(P_{i+1} - P_i\right)\right]^{\frac{1}{2}}, \qquad (12)$$

where the sign is dependent on the direction in which the shock is being reflected. Eqs. 2 and 3 can then be used to find $u_{s\,i+1}$ and E_{i+1} respectively. Once the thermodynamic parameters have been calculated for first shock using the Rankine-Hugoniot equations, the parameters can be updated for subsequent shocks using the equations above. Isotropic compression can be approximated by calculating the results for a series of small small shocks.

CALCULATION OF TEMPERATURE INCREASES

To calculate the increase in temperature, from Meyers [1] we have-

$$\left(\frac{dE}{dV}\right) = C_v\left(\frac{dT}{dV}\right) + \frac{\gamma T}{V}C_v - P \qquad (13)$$

$$\left(\frac{dT}{dV}\right) + \frac{\gamma}{V} = \left[\left(\frac{dE}{dV}\right) + P\right]/C_v, \qquad (14)$$

where C_v is the specific heat at constant volume. This can be solved using an integrating factor-

$$\mu(V) = \exp\left(\int \frac{\gamma}{V}dV\right). \qquad (15)$$

We therefore have-

$$\mu(V)\left[\left(\frac{dT}{dV}\right) + \frac{\gamma T}{V}\right] = \mu(V)\left\{\left[\left(\frac{dE}{dV}\right) + P\right]/C_v\right\} \qquad (16)$$

$$\frac{d}{dV}\left[T\mu(V) + c_1\right] = \mu(V)\left\{\left[\left(\frac{dE}{dV}\right) + P\right]/C_v\right\} \qquad (17)$$

$$T_{i+1}\mu(V_{i+1}) = \int_{V_i}^{V_{i+1}} \mu(V)\left\{\left[\left(\frac{dE}{dV}\right) + P\right]/C_v\right\}dV - c_1 \qquad (18)$$

$$T = \frac{1}{\mu(V_{i+1})} \times \left[\int_{V_i}^{V_{i+1}} \mu(V)\left\{\left[\left(\frac{dE}{dV}\right) + P\right]/C_v\right\}dV - c_1\right]. \qquad (19)$$

Since $T_{i+1}(V_i) = T_i$, we find that $c_1 = -T_i\mu(V_i)$, so we have-

$$T_{i+1} = T_i\frac{\mu(V_i)}{\mu(V_{i+1})} + \frac{1}{\mu(V_{i+1})}\int_{V_i}^{V_{i+1}} \mu(V)\left\{\left[\left(\frac{dE}{dV}\right) + P\right]/C_v\right\}dV. \qquad (20)$$

From integrating by parts, we have-

$$\int \mu\frac{dE}{dV}dV = \mu E - \int \frac{d\mu}{dV}EdV \qquad (21)$$

From Eq 15, we find that $\frac{d\mu}{dV} = \frac{\gamma}{V}\mu$. Substituting this into Equation 20, we find-

$$T_{i+1} = T_i\frac{\mu(V_i)}{\mu(V_{i+1})} + \frac{1}{\mu(V_{i+1})C_v}\left\{\left[\mu(V_{i+1})E(V_{i+1}) - \mu(V_i)E(V_i)\right] + \int_{V_i}^{V_{i+1}} \mu(V)\left[P - \frac{\gamma}{V}E(V)\right]dV\right\}. \qquad (22)$$

If we let $\gamma = \gamma_0\left(\frac{V}{V_0}\right)^q$, then we find $\mu(V) = \exp\left[\frac{1}{q}\left(\frac{V}{V_0}\right)^q\right]$. Substituting this into Eq 22, we find-

$$T_{i+1} = \left(T_i - \frac{E(V_i)}{C_v}\right)\exp\left[\frac{\gamma_0}{q}\left\{\left(\frac{V_i}{V_0}\right)^q - \left(\frac{V_{i+1}}{V_0}\right)^q\right\}\right] + \frac{E(V_{i+1})}{C_v} + \frac{\exp\left[-\frac{\gamma_0}{q}\left(\frac{V_{i+1}}{V_0}\right)^q\right]}{C_v} \times \int_{V_i}^{V_{i+1}} \exp\left[\frac{\gamma_0}{q}\left(\frac{V}{V_0}\right)^q\right]\left[P - \frac{\gamma_0 V^{q-1}}{V_0^q}E(V)\right]dV. \qquad (23)$$

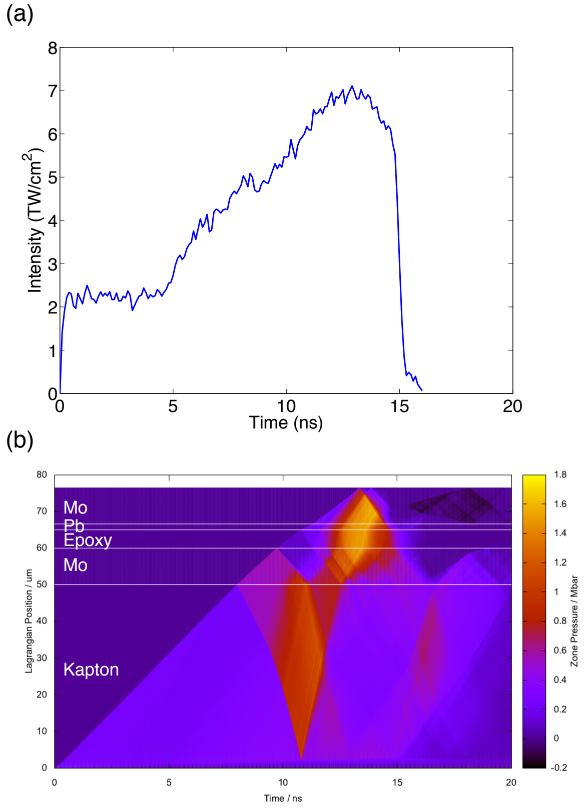

FIG. 1. (a) A typical laser pulse shape. (b) A HYADES simulation using the experimental laser profile.

For a Hugoniot shock process, we have-

$$E_{i+1} - E_i = \frac{1}{2}\left(P_{i+1} + P_i\right)\left(V_i - V_{i+1}\right) \tag{24}$$

Substituting this into Equation 22, we find-

$$\begin{aligned} T_{i+1} = \left(T_i - \frac{E(V_i)}{C_v}\right) \exp\left[\frac{\gamma_0}{q}\left\{\left(\frac{V_i}{V_0}\right)^q - \left(\frac{V_{i+1}}{V_0}\right)^q\right\}\right] \\ + \frac{(P(V_{i+1}) + P(V_i))(V_i - V_{i+1}) + 2E(V_i)}{2C_v} \\ + \frac{\exp\left[-\frac{\gamma_0}{q}\left(\frac{V_{i+1}}{V_0}\right)^q\right]}{2C_v} \int_{V_i}^{V_{i+1}} \exp\left[\frac{\gamma_0}{q}\left(\frac{V}{V_0}\right)^q\right] \\ \left[2P(V) - \frac{\gamma_0 V^{q-1}}{V_0^q}\left\{(P(V) + P(V_i))(V_i - V) + E(V_i)\right\}\right] dV, \end{aligned} \tag{25}$$

which for $V_i = V_0,\ P(V_i) = 0,\ E(V_i) = 0$ and $q = 1$

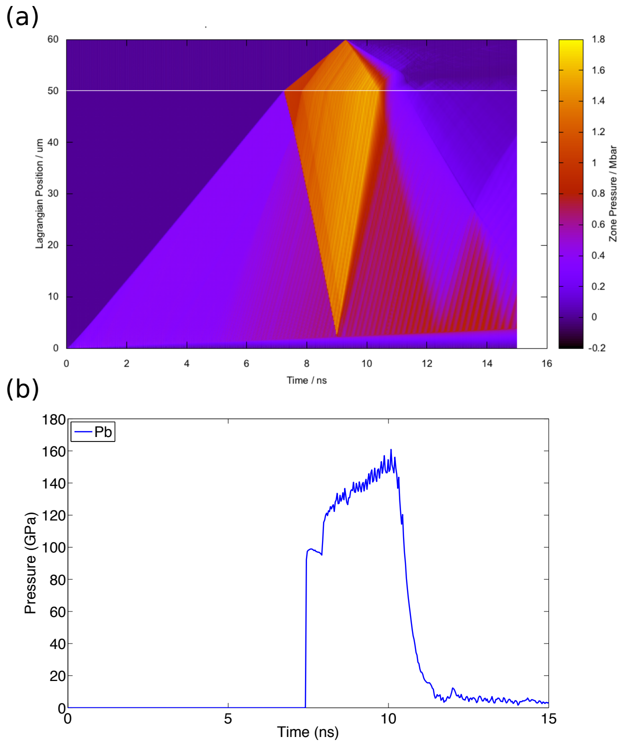

FIG. 2. (a) HYADES simulation and (b) Pb pressure profile of a Kapton-Pb target using the same laser pulse as Figure 1. The pulse was scaled up by a factor of 1.85 to achieve the same final pressure.

simplifies to the equation given in Meyers-

$$\begin{aligned} T_{i+1} = T_i \exp\left[\gamma_0\left(\frac{V_0 - V_{i+1}}{V_0}\right)\right] + \frac{P(V_{i+1})(V_0 - V_{i+1})}{2C_v} \\ + \frac{\exp\left[(-\gamma_0/V_0)\, V_{i+1}\right]}{2C_v} \int_{V_0}^{V_{i+1}} \exp\left[(-\gamma_0/V_0)\, V_{i+1}\right] \\ \times \left[2P(V) - \frac{\gamma_0}{V_0} P(V_{i+1})(V_0 - V)\right] dV. \end{aligned} \tag{26}$$

This can be combined with the results of the previous section to find the temperature of the sample after each shock. The parameters used to model each layer in the target are shown in Table I, as well as the SESAME equations of state used for the HYADES simulation. The specific heat of Pb was taken to be 124 J/kg/K.

HYDROCODE SIMULATION

To better understand how the target behaved during the experiment, the laser pulses recorded during the experiment (Figure S1a) were used to generate pressure profiles for HYADES simulations. The ramped laser

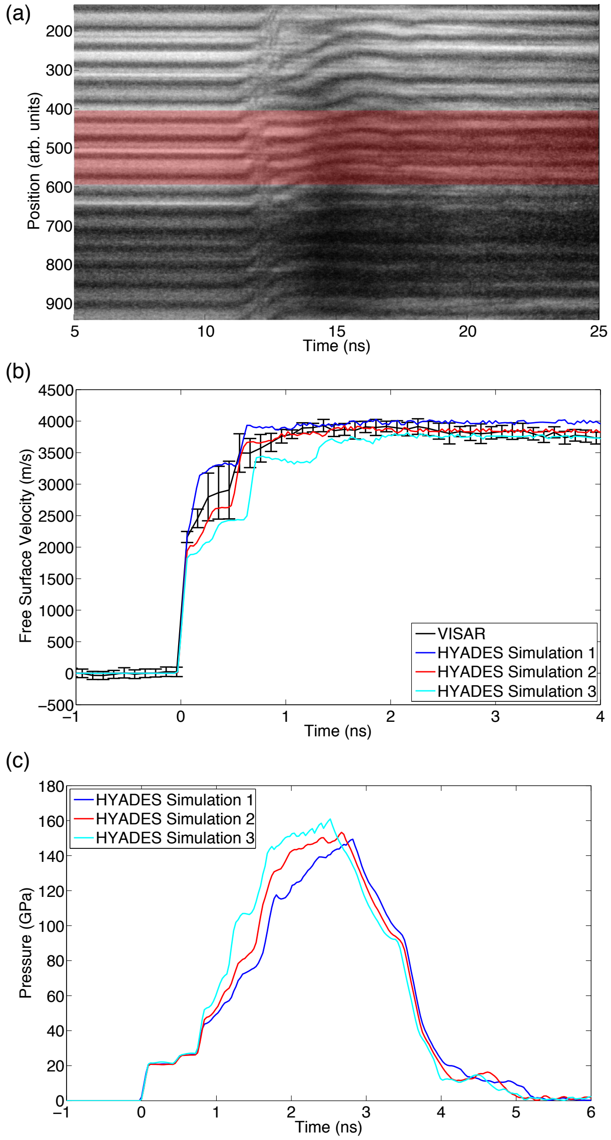

FIG. 3. (a) A typical VISAR image from one of the streak cameras. The area highlighted in red corresponds to the position of the 35 μm-diameter XFEL beam. The free surface velocity (b) and Pb pressure (c) from three HYADES simulations using the laser intensity profile shown in Figure S1b. The laser profile was scaled to match the free surface velocity recorded by VISAR (simulation 2) as well as the upper (simulation 1) and lower (simulation 3) bounds. This yielded a maximum pressure in the Pb of 150 ± 10 GPa.

pulse yields an additional compression at later times, resulting in a cooler compression path.

However, this poses a problem as a ramped pressure profile could explain why the Pb sample does not melt, rather than the ringing within the target. To demonstrate this is not the case, the same laser pulse was used in a simulation of a Kapton-Pb target, shown in Figure S2. Note that since the 'ring up' target is more efficient, the laser pulse energy had to be scaled up by a factor of 1.85 to generate the same final pressure. This resulted in an initial shock in the Pb sample of ~ 100 GPa before ramping up to 150 GPa. The temperature on the Hugoniot at 100 GPa is $\sim$ 7000K and since this is above the melt curve at 150 GPa, the 'ring up' target must have resulted in significantly reducing the temperature of the sample in order for it to have remained solid.

PRESSURE DETERMINATION

To determine the pressure reached in the experiment, the rear surface velocity as a function of time was monitored using velocity interferometry (VISAR). This can be compared to HYADES simulations, similar to those shown in Figure S1. By varying the intensity the laser pulse, we find the final pressure to be 150 ± 10 GPa. This can also be used to find the pressure of the first shock in the Pb. However, the error on this measurement is dominated by the quality of the VISAR fringes in this region, which can be tracked to within $\pm 25\%$ of a fringe, corresponding to $\pm 250\text{ms}^{-1}$. This results in a measurement of the first shock pressure of 19.5 ± 6.5 GPa. These two errors, as well as the error in q for Pb, which is taken to be ± 1 are then used to generate the errors in pressure and temperature in Figure 6.

PROCESSING OF DIFFRACTION DATA

X-ray diffraction patterns of CeO_2 and LaB_6 were recorded to find the positions and tilts of the CSPAD detectors. Using this information, the raw diffraction images (once background subtracted) were warped into spherical coordinates (shown in Figure S4a). To find the pixels that need to masked to remove the Mo spots, first the warped image have a moving average filter applied in the azimuthal direction. The image is then split up into 20 vertical strips. Each pair of adjacent strips are put together to form an image and for each row, the 10th percentile is calculated. Each pixel that is above twice the 10th percentile of its row is tagged to be added to the mask. By repeating this process for each set of adjacent strips, we are able to form a mask to remove the contribution from the Mo spots (shown in Figure S4b). To further improve the quality of the image, every pixel within 5 pixels of those originally tagged are also added to the mask (shown in Figure S4c). To produce lineouts (shown in Figure S4d), the masks were applied to the original diffraction data and then integrated along the azimuthal direction.

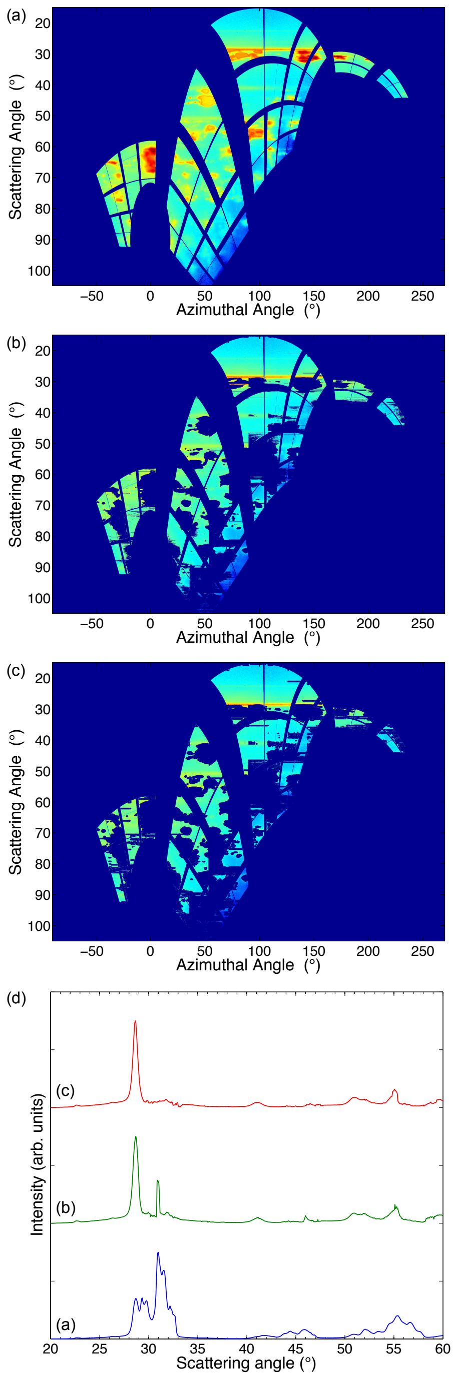

FIG. 4. The diffraction data warped in spherical coordinates, shown with (a) no mask, (b) only pixels tagged as twice the 10th percentile masked and (c) also all pixels within 5 pixels of those originally tagged masked. (d) The effect of the different masks on the azimuthally integrated lineouts.

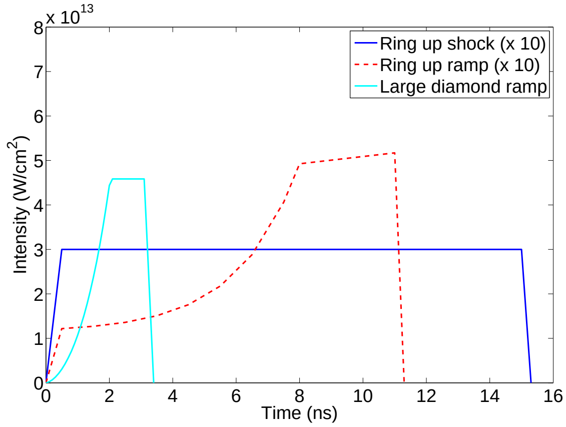

FIG. 5. Examples of the laser pulses used in the efficiency comparison. At a similar pressure, the peak intensity of the pulses used for the 'ring-up' targets are approximately an order of magnitude lower in peak intensity than for diamond sandwich targets.

COMPARISON OF LASER PULSE EFFICIENCIES

Figure S5 shows the intensity profile of the laser pulses used for the four different simulations shown in Figure 7. Each simulation has a peak pressure of approximately 240 GPa.

* d.mcgonegle1@physics.ox.ac.uk

[1] M. A. Meyers, *Dynamic Behavior of Materials* (John Wiley & Sons, Inc., Hoboken, NJ, USA, 1994).
